# Effects of CB2 and TRPV1 receptors’ stimulation in pediatric acute T-lymphoblastic leukemia

**DOI:** 10.18632/oncotarget.25052

**Published:** 2018-04-20

**Authors:** Francesca Punzo, Iolanda Manzo, Chiara Tortora, Elvira Pota, Velia D’ Angelo, Giulia Bellini, Alessandra Di Paola, Federica Verace, Fiorina Casale, Francesca Rossi

**Affiliations:** ^1^ Department of Women, Child and General and Specialist Surgery, University of Campania “Luigi Vanvitelli”, Naples 80138, Italy; ^2^ Department of Experimental Medicine, Division of Pharmacology “Leonardo Donatelli”, University of Campania “Luigi Vanvitelli”, Naples 80138, Italy

**Keywords:** oncogene, MYB, SKI, transcriptional regulation, acute myeloid leukemia

## Abstract

T-Acute Lymphoblastic Leukemia (T-ALL) is less frequent than B-ALL, but it has poorer outcome. For this reason new therapeutic approaches are needed to treat this malignancy.

The Endocannabinoid/Endovanilloid (EC/EV) system has been proposed as possible target to treat several malignancies, including lymphoblastic diseases. The EC/EV system is composed of two G-Protein Coupled Receptors (CB1 and CB2), the Transient Potential Vanilloid 1 (TRPV1) channel, their endogenous and exogenous ligands and enzymes. CB1 is expressed mainly in central nervous system while CB2 predominantly on immune and peripheral cells, therefore we chose to selectively stimulate CB2 and TRPV1.

We treated T-ALL lymphoblasts derived from 4 patients and Jurkat cells with a selective agonist at CB2 receptor: JWH-133 [100 nM] and an agonist at TRPV1 calcium channel: RTX [5 uM] at 6, 12 and 24 hours. We analyzed the effect on apoptosis and Cell Cycle Progression by a cytofluorimetric assays and evaluated the expression level of several target genes (Caspase 3, Bax, Bcl-2, AKT, ERK, PTEN, Notch-1, CDK2, p53) involved in cell survival and apoptosis, by Real-Time PCR and Western Blotting.

We observed a pro-apoptotic, anti-proliferative effect of these compounds in both primary lymphoblasts obtained from patients with T-ALL and in Jurkat cell line. Our results show that both CB2 stimulation and TRPV1 activation, can increase the apoptosis *in vitro*, interfere with cell cycle progression and reduce cell proliferation, indicating that a new therapeutic approach to T-cell ALL might be possible by modulating CB2 and TRPV1 receptors.

## INTRODUCTION

Leukemias encompass 30% of all pediatric cancers, and acute lymphoblastic leukemia (ALL) is the most common leukemia in the pediatric population; it comprises 75% of all pediatric leukemia cases [1, 2]. Childhood ALL incidence is 3–4 cases per 100,000 in under 15-year-old children and it usually occurs when the child is between 2 and 5 years old. ALL is an heterogeneous disease: subtypes differ with regard to biological, cellular and molecular characteristics, response to therapy and risk of relapse, and are associated with different outcomes [3–6]. T-ALL constitutes approximately 15% of pediatric ALL but shows a poorer outcome compared to B-ALL [7]. In fact, children with T-ALL tend to experience more induction failures and extramedullary relapses than their B-ALL counterparts and frequently present with unfavorable clinical features, such as male gender, older age, high white blood cell count (WBC), bulky extramedullary disease, and CNS involvement [8, 9]. In spite of improved survival rates obtained with risk-adjusted therapy, 25% of T-ALL patients have little or no expectancy of cure. Hence, identification of new prognostic markers and development of new therapeutic targets remain a critical task to help children with T-ALL. In the last decade, several studies proposed cannabinoids as antineoplastic drugs to treat malignancies of the immune system [10–16]. “Cannabinoid” is the collective term for a group of chemical compounds that derive from the *Cannabis* plant, like as synthetic or endogenous (endocannabinoids) analogues, and that interact with specific receptors: cannabinoid receptor type 1 (CB1), cannabinoid receptor type 2 (CB2), transient receptor potential vanilloid type 1 (TRPV1). Generally, CB1 signaling mediates neuromodulatory activities (CB1 receptors are expressed at high levels in CNS), and CB2 signaling mostly mediates immunomodulatory activities of these compounds (CB2 receptors are primarily expressed on immune and peripheral cells) [17, 18]. TRPV1 has been described as an additional receptor target for several cannabinoids such as Anandamide which is capable of binding both Cannabinoids and Vanilloid receptors [19, 20]. When TRPV1 channel proteins are activated, they induce massive calcium intake in the cell [21]. Intracellular calcium overload mediate cell apoptosis through different mechanism interfering with cell energy production and metabolism, therefore also drugs acting on the TRPV receptors, potentially can act as target to reduce cell proliferation and survival in cancer [22, 23]. Evidences on the cross-talk between CB2 receptors and TRPV1 channels have been demonstrated [24–27]. Cannabinoid receptors have been shown to modulate several signaling pathways involved in the control of cell proliferation and survival [28–31]. Moreover we have recently demonstrated an anti-proliferative, pro-apoptotic and anti-invasive effect induced by EC/EV compounds in human osteosarcoma [32].

The ability to regulate the apoptotic process upon their activation, the selective presence of the CB2 and the TRPV1 on immune system cells, and their ability not to induce psychotropic effects, suggest these receptors as new possible pharmacological targets for those diseases affecting the immune system cells. Based on these evidences, we investigated for the first time the expression of CB2 and TRPV1 receptors in primary lymphoblast cultures deriving from 4 T-ALL patients and in Jurkat cell line. We evaluated the effects of two agonists of the EC/EV system (RTX selective on TRPV1 receptors and JWH-133 selective over CB2 receptors) at different times of exposure in these cells, analyzing the effects on cell survival, cell cycle progression and apoptosis. We observed an anti-proliferative, pro-apoptotic effect induced by these EC/EV compounds in T-ALL patients and Jurkat cells.

## RESULTS

### Jurkat and T-ALL cells express EC/EV system

We first evaluated the expression of CB2 and TRPV1 in untreated Jurkat cell line and primary patient's lymphoblasts, to verify the presence of the receptors we were going to stimulate. The result demonstrated the presence of mature mRNA for CB2 and TRPV1 receptors (Supplementary Figure 1).

### Effect of EC/EV compounds in Jurkat and T-ALL cells on Apoptosis

**Figure 1:**
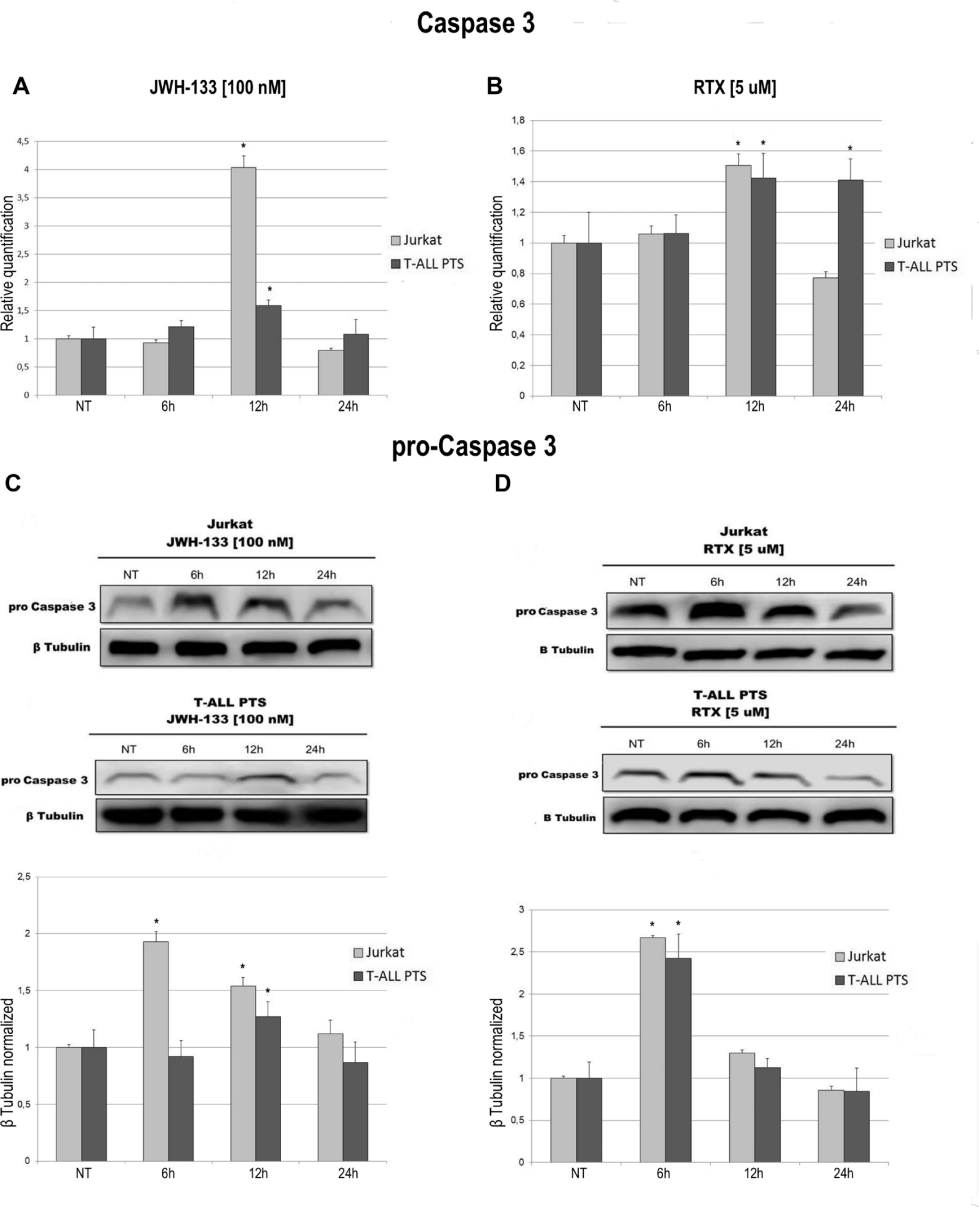
Effects of JWH-133 and RTX treatments on Caspase-3 mRNA expression levels and on Pro-Caspase 3 protein expression levels Caspase-3 mRNA expression levels in Jurkat cell line and in T-ALL patients’ lymphoblasts derived from 4 patients were determined by Q-PCR after JWH-133 [100 nM] treatment (**A**) and RTX [5 uM] (**B**) treatments at 6 h, 12 h and 24 h of exposure. Results were normalized for the housekeeping gene β-actin and were showed as mean ± SD of three independent experiments in Jurkat cells (performed in technical triplicate), and as mean ± SD of four single experiments on each one of the 4 patients (performed in technical triplicate). A *t*-test has been used to evaluate statistical differences in Caspase-3 mRNA expression among groups. ^*^indicates *p* ≤ 0.05 compared to the untreated control (NT). Pro-Caspase 3 protein expression levels in Jurkat cell line and in T-ALL patients’ lymphoblasts, were determined by Western Blot, starting from 15 μg of total lysates after JWH-133 [100 nM] (**C**) and RTX [5 uM] (**D**) treatments at 6 h, 12 h and 24 h of exposure. The images show the most representative blots. The proteins were detected using Image Studio Digit software and the intensity ratios of immunoblots compared to the one of the untreated control, taken as 1(arbitrary unit), were quantified after normalizing with respective loading controls for the housekeeping protein β-tubulin. The graphs show the relative quantification for pro-Caspase-3, represented as mean ± SD of two independent experiments for Jurkat cells and as mean ± SD of four single experiments performed on each one of the 4 patients. A *t*-test has been used to evaluate the statistical differences in protein expression levels. ^*^ indicates *p* ≤ 0.05 compared to the untreated control (NT).

**Figure 2:**
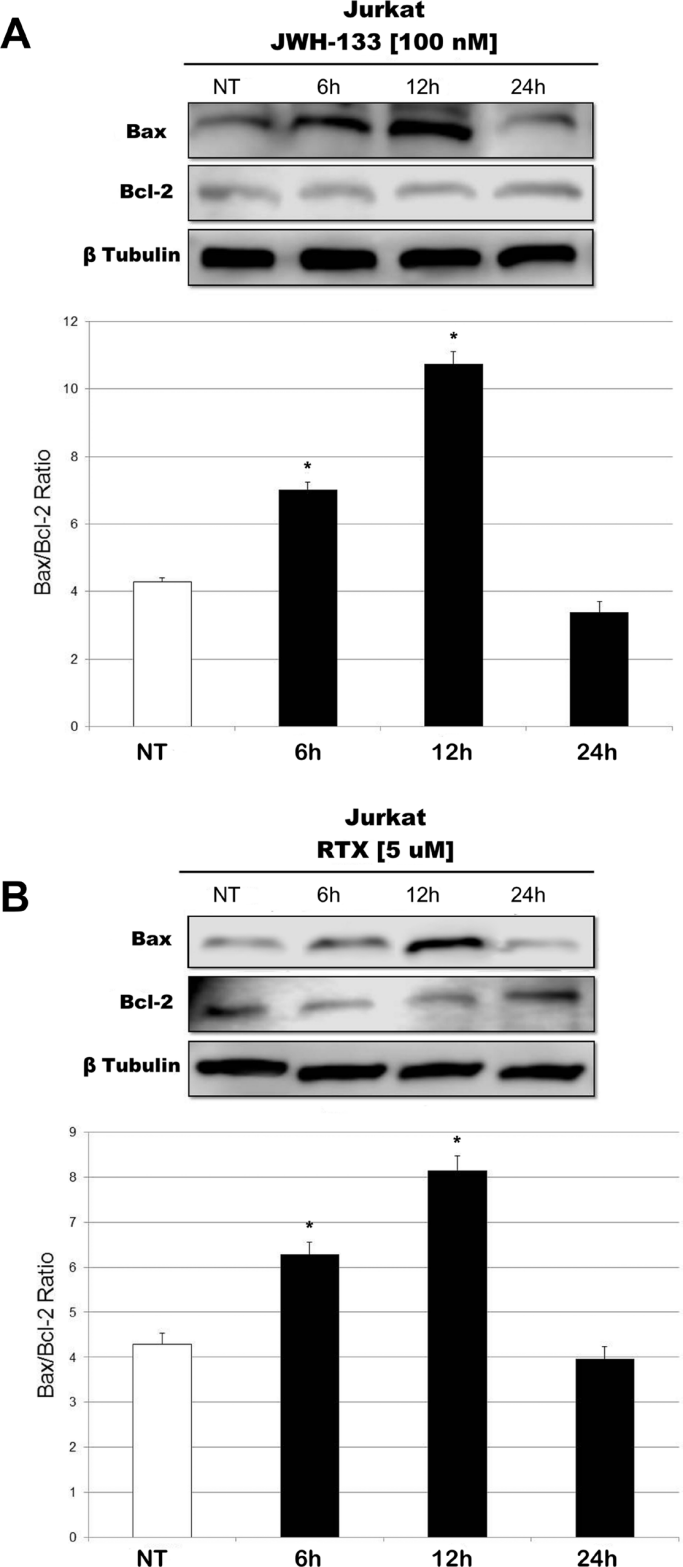
Effects of JWH-133 and RTX treatments on Bax/Bcl-2 ratio, in Jurkat cell line Bax and Bcl-2 protein expression levels in Jurkat cell line, determined by Western Blot, starting from 15 μg of total lysates after JWH-133 [100 nM] (**A**) and RTX [5 uM] (**B**) treatments at 6 h, 12 h and 24 h of exposure. The results were normalized for the housekeeping protein β-tubulin. The most representative images are displayed. The graphs represent the ratio between Bax and Bcl-2 as the mean and ± S.D. from two experiments. A *t*-test has been used to evaluate the statistical differences in protein expression levels. ^*^indicates *p* ≤ 0.05 compared to the untreated control (NT).

We observed an effect on apoptosis in both patients’ and Jurkat cells after Vanilloid and Cannabinoid stimulation with selective agonists (RTX and JWH-133) (Table 1 and Supplementary Figure 2). Both JWH-133 [100 nM] and RTX [5 uM] increased apoptosis in all samples and at all time points, and the difference at 6 h and 12 h was statistically significant in both cell types, while at 24 h it remained statistically relevant only in Patients’ cells, compared to the non-treated cell line. To evaluate the possible molecular mechanism through which EC/EV drugs act on apoptosis, we analyzed the expression levels of Caspase-3 after JWH-133 [100 nM] (Figure 1A) and RTX [5 μM] (Figure 1B) treatments by Real Time PCR and Western Blotting (Figure 1C, 1D). Both compounds (JWH-133 and RTX) induced a significant increase of Caspase-3 expression in T-ALL patients and Jurkat cells (Figure 1A, 1B) at mRNA level, where we evaluated the Caspase 3 mRNA and also by Western Blot where the Pro-Caspase 3 levels were examined (Figure 1C, 1D). Moreover we evaluated also Bax/Bcl-2 ratio (only in Jurkat cells) in order to add more evidences on the effect of these compounds on Apoptosis. The Ratio is increased in a statistically significant level following administration of both JWH-133 [100 nM] and RTX [5 μM] (Figure 2A, 2B).

**Table 1:** Percentage of total apoptotic cells in Jurkat cell line and in T-ALL patients’ lymphoblasts after JWH-133 and RTX treatments

*Jurkat*	6 h (%)	12 h(%)	24 h(%)
**NT**	22.8 ± 2.3	15.1 ± 2.4	26.5 ± 2.7
**JWH-133 [100 nM]**	46.9 ± 2.3^*^	28.5 ± 2.4^*^	33.3 ± 2.5^*^
**RTX [5 μM]**	52.3 ± 2.2^*^	42.5 ± 2.4^*^	30.8 ± 2.7
***ALL T PTS***	**6 h (%)**	**12 h (%)**	**24 h (%)**
**NT**	13.8 ± 4.1	20.9 ± 4.9	14.4 ± 4.8
**JWH-133 [100 nM]**	32.9 ± 5.4^*^	30.4 ± 3.1^*^	26.8 ± 4.9^*^
**RTX [5 μM]**	30.2 ± 4.2^*^	32.5 ± 4.7^*^	26.5 ± 4.8^*^

Analysis of cell apoptosis by Annexin-V and PI double-stained in Jurkat cells line and T-ALL patients’ lymphoblasts derived from 4 patients induced by JWH-133 [100 nM] and RTX [5 uM], after 6 h, 12 h and 24 h of exposure. The results are presented as the mean percentage ± SD of three independent experiments for Jurkat cells and as mean percentage ± SD of 4 single experiments on each one of the 4 patients. The results were analyzed by one-way analysis of variance (ANOVA). ^*^indicates *p* ≤ 0.05 compared to the untreated control (NT).

### Effects of EC/EV compounds in Jurkat and T-ALL cells on cell proliferation

To evaluate the possible molecular mechanisms through which EC/EV selective agonists act on cell proliferation, we analysed the expression levels of a series of genes known to be involved in cell proliferation such as AKT (or PKB, Protein Kinase B), ERK (Mitogen-Activated-Protein Kinase 1), PTEN (Phosphatase and Tensin Homolog), NOTCH-1 and Tumor Protein p53.

**Figure 3:**
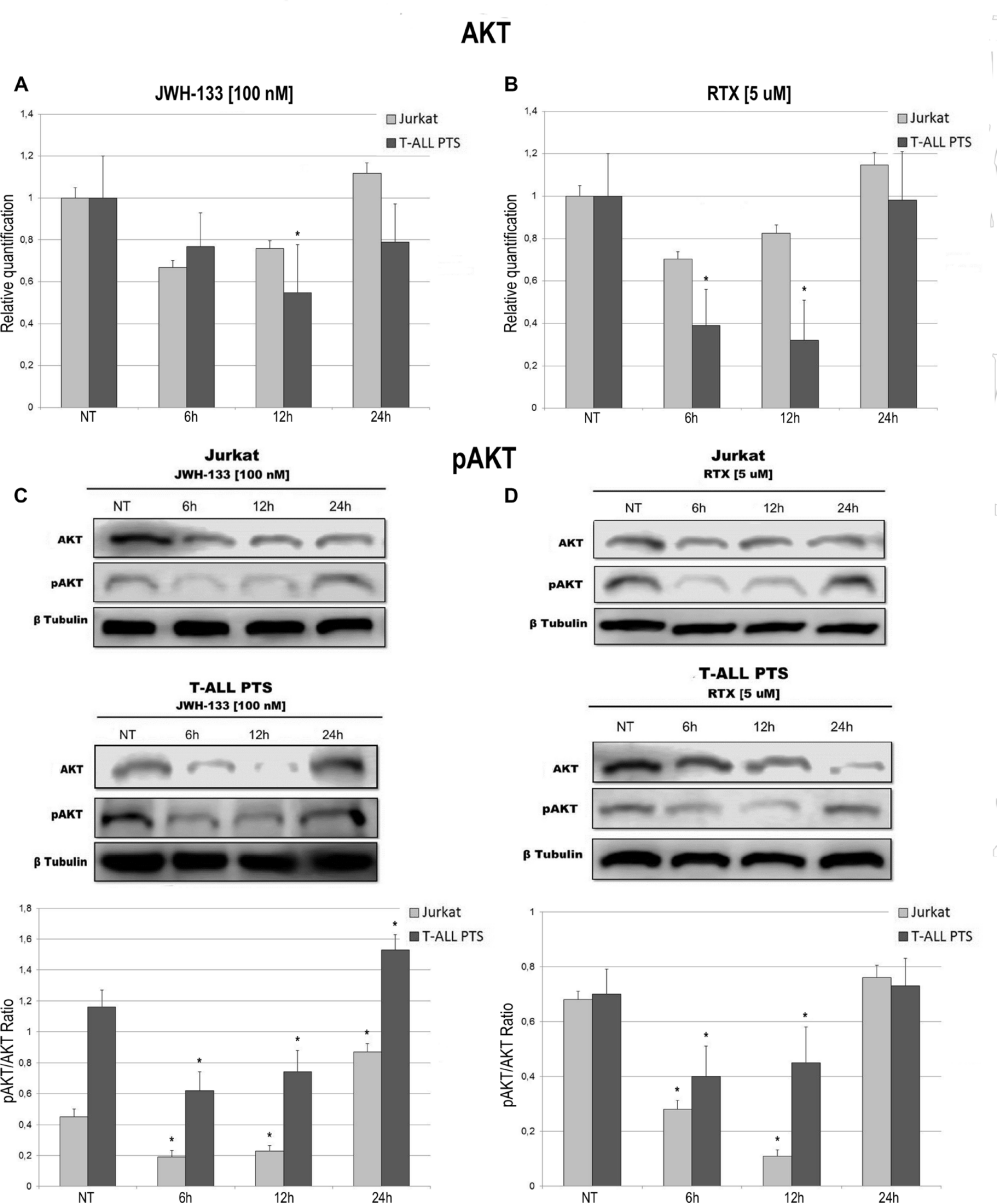
Effects of JWH-133 and RTX treatments on AKT mRNA expression levels and pAKT protein expression levels AKT mRNA expression levels in Jurkat cell line and in T-ALL patients’ lymphoblasts derived from 4 patients were determined by Q-PCR after JWH-133 [100 nM] (**A**) and RTX [5 uM] (**B**) treatments after 6 h, 12 h and 24 h of exposure. Results were normalized for the housekeeping gene β-actin and were showed as mean ± SD of three independent experiments for Jurkat cells (performed in technical triplicate), and as mean ± SD of four single experiments on each one of the 4 patients (performed in technical triplicate). A *t*-test has been used to evaluate statistical differences in AKT expression among groups. ^*^indicates *p* ≤ 0.05 compared to the untreated control (NT). AKT and pAKT protein expression levels in Jurkat cell line and in T-ALL patients’ lymphoblasts, were determined by Western Blot, starting from 15 μg of total lysates after JWH-133 [100 nM] (**C**) and RTX [5 uM] (**D**) treatments after 6 h, 12 h and 24 h of exposure. The images show the most representative blots. The proteins were detected using Image Studio Digit software and the intensity ratios of immunoblots compared to the one of the untreated control, taken as 1(arbitrary unit), were quantified after normalizing with respective loading controls for the housekeeping protein β-tubulin. The graphs show the ratio between pAKT and AKT as the mean ± SD of two independent experiments for Jurkat cells and as mean ± SD of four single experiments on each one of the 4 patients. A *t*-test has been used to evaluate the statistical differences in protein expression levels. ^*^ indicates *p* ≤ 0.05 compared to the untreated control (NT).

**Figure 4:**
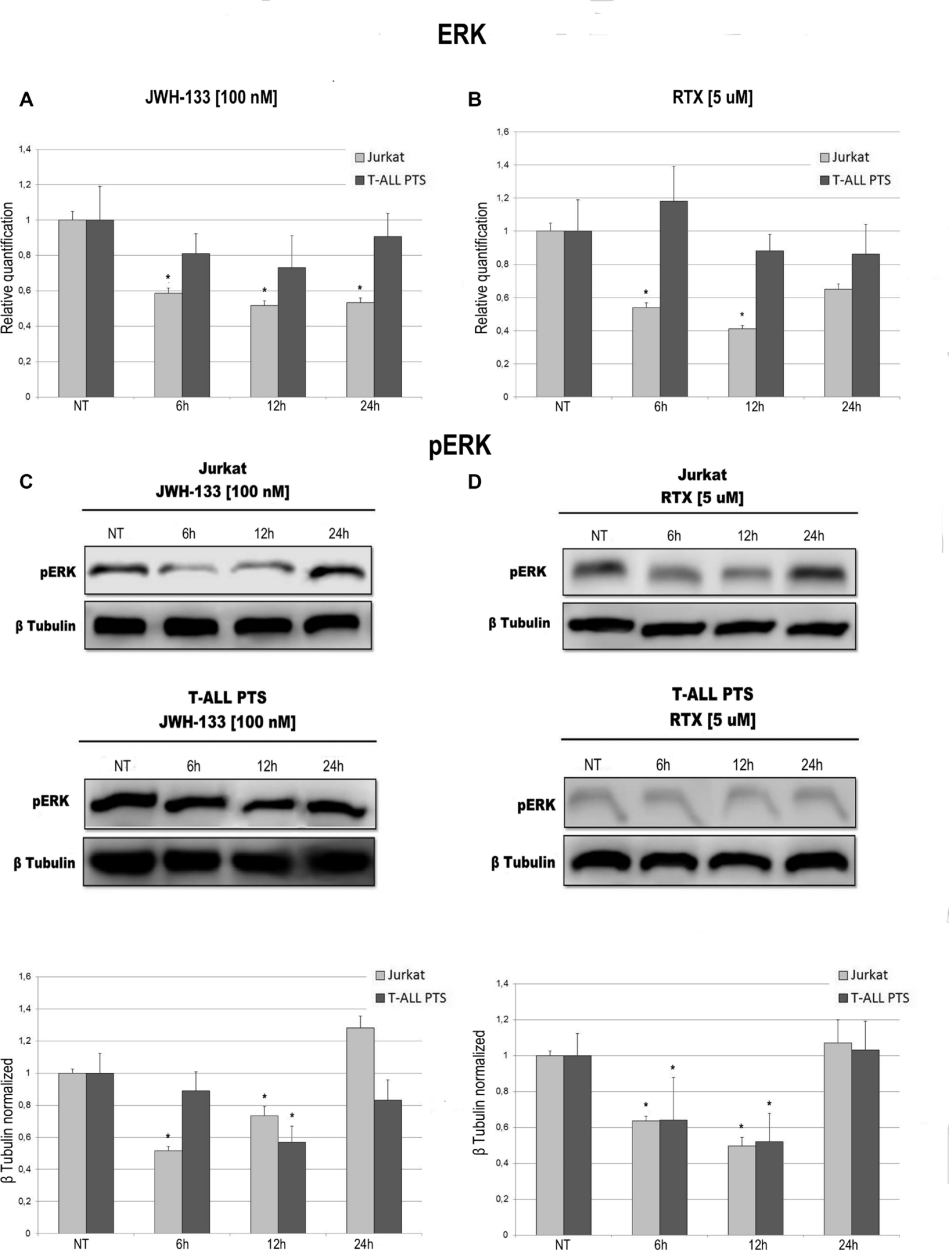
Effects of JWH-133 and RTX treatments on ERK mRNA expression levels and pERK protein expression levels ERK mRNA expression levels in Jurkat cell line and in T-ALL patients’ lymphoblasts derived from 4 patients were determined by Q-PCR after JWH-133 [100 nM] (**A**) and RTX [5 uM] (**B**) treatments at 6 h, 12 h and 24 h of exposure. Results were normalized for the housekeeping gene β-actin and were showed as mean ± SD of three independent experiments for Jurkat cells (performed in technical triplicate), and as mean ± SD of four single experiments on each one of the 4 patients (performed in technical triplicate). A *t*-test has been used to evaluate statistical differences in ERK expression among groups. ^*^indicates *p* ≤ 0.05 compared to the untreated control (NT). pERK protein expression levels in Jurkat cell line and in T-ALL patients’ lymphoblasts, were determined by Western Blot, starting from 15 μg of total lysates after JWH-133 [100 nM] (**C**) and RTX [5 uM] (**D**) treatments after 6 h, 12 h and 24 h of exposure. Images show the most representative blots. The proteins were detected using Image Studio Digits software and the intensity ratios of immunoblots compared to the untreated control, taken as 1(arbitrary unit), were quantified after normalizing with respective loading controls for the housekeeping protein β-tubulin. The results were normalized for the housekeeping protein β-tubulin. The graphs represent the relative quantification for pERK expression as mean ± SD from two independent experiments for Jurkat cells and as mean ± SD of four single experiments on each one of the 4 patients. A *t*-test has been used to evaluate the statistical differences in protein expression levels. ^*^indicates *p* ≤ 0.05 compared to the untreated control (NT).

Total AKT levels measured by Real Time PCR are reduced in Jurkat and T-ALL patient's lymphoblasts by both compounds (JWH-133 and RTX) (Figure 3A, 3B). The phosphorylated form of AKT was remarkably reduced, in Jurkat cells and in T-ALL patients’ cells, 6 h and 12 h after JWH-133 [100 nM] (Figure 3C) and RTX [5 μM] (Figure 3D) exposure as shown by Western Blot analysis. To quantify the reduction of the active form of Akt we calculated the pAkt/Akt ratio as shown in the graphs of Figure 3C, 3D. Moreover, JWH-133 [100 nM] treatment results in a significant decrease of ERK mRNA levels at all time points (6 h, 12 h and 24 h) in Jurkat cells, while in patients the decrease is not statistically significant (Figure 4A). RTX [5 μM] treatment induced a significant decrease of ERK mRNA expression in Jurkat cell lines after 6 h and 12 h of exposure as compared to respective control, while in patients’ cells there are not significant changes (Figure 4B) whereas when analyzing the active form of the protein by Western Blot, pERK resulted reduced in a statistically significant manner, in both Jurkat and T-ALL patient's cells by both treatments (JWH-133 and RTX) (Figure 4C, 4D).

**Figure 5:**
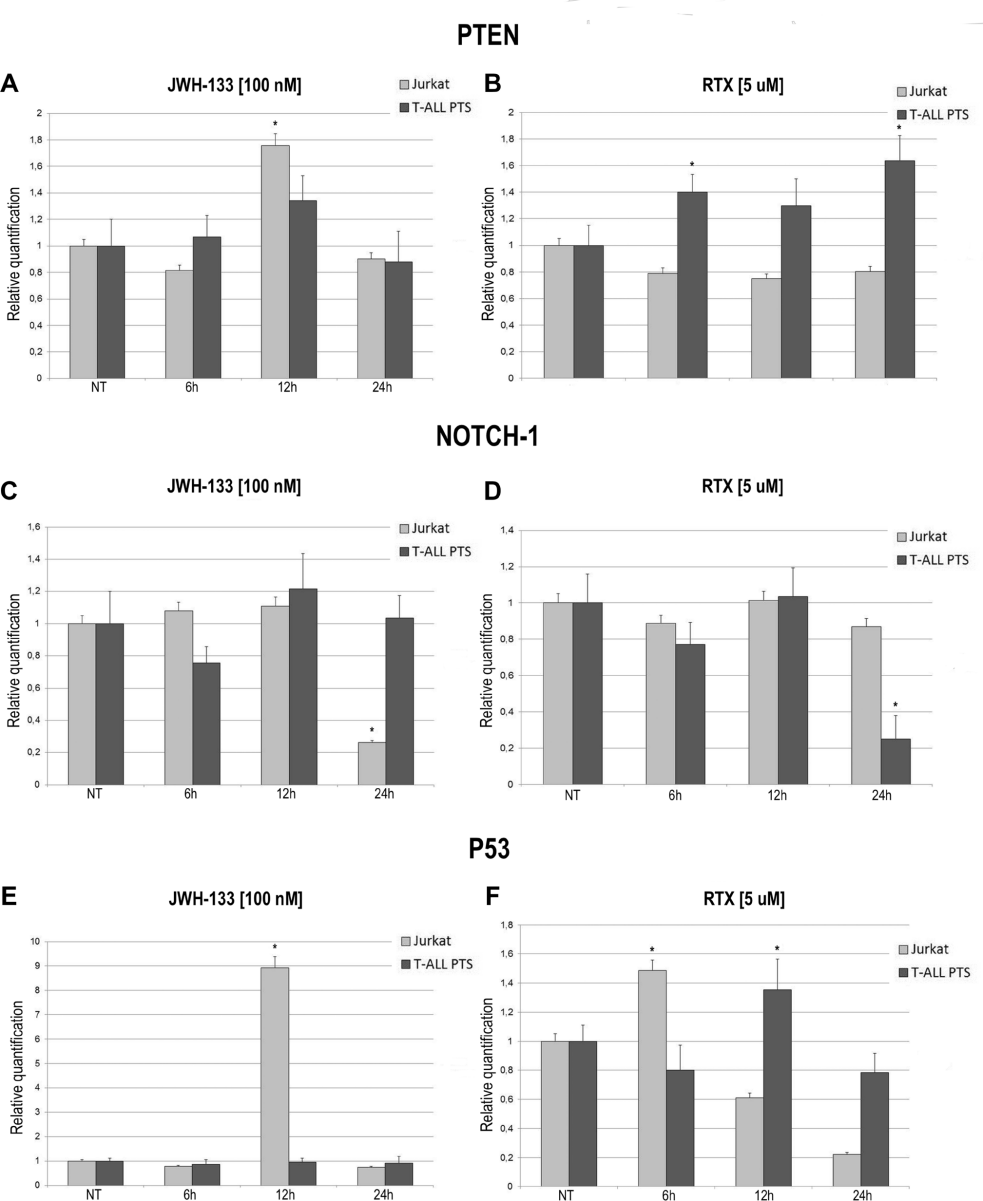
Effects of JWH-133 and RTX treatments on PTEN, NOTCH-1 and P53 mRNA expression levels PTEN, NOTCH-1 and P53 mRNA expression levels in Jurkat cell line and in T-ALL patients’ lymphoblasts derived from 4 patients were determined by Q-PCR after JWH-133 [100 nM] (**A**, **C**, **E**) and RTX [5uM] (**B**, **D**, **F**) treatments at 6 h, 12 h and 24 h of exposure. Results were normalized for the housekeeping gene β-actin and were showed as mean ± SD of three independent experiments for Jurkat cells (performed in technical triplicate), and as mean ± SD of four single experiments on each one of the 4 patients (performed in technical triplicate). A *t*-test has been used to evaluate statistical differences in PTEN, NOTCH-1 and P53 expression among groups. ^*^indicates *p* ≤ 0.05 compared to the untreated control (NT).

The expression levels of PTEN, NOTCH-1 and p53 after JWH-133 [100 nM] and RTX [5 μM] treatments, was evaluated by Real Time PCR (Figure 5). JWH-133 treatment induced a significant increase of PTEN mRNA levels in the Jurkat cells while in patients the increase is still visible but the variation is not statistically significant (Figure 5A). On the other hand RTX induced a significant increase of PTEN mRNA levels only in T-ALL patients cells whereas in Jurkat cell line we did not observed any effect (Figure 5B). Expression level of NOTCH-1 decreases significantly only after treatment with JWH-133 in Jurkat and with RTX in T-ALL Patients (Figure 5C, 5D) while mRNA levels of p53 resulted dramatically increased by JWH-133 [100 nM] and RTX [5 μM] treatments in Jurkat cells. In T-ALL patients p53 mRNA levels displayed a statistically significant increase only after Vanilloid stimulation (Figure 5E, 5F).

### Effect of EC/EV compounds in Jurkat and T-ALL cells on cell cycle progression

**Figure 6:**
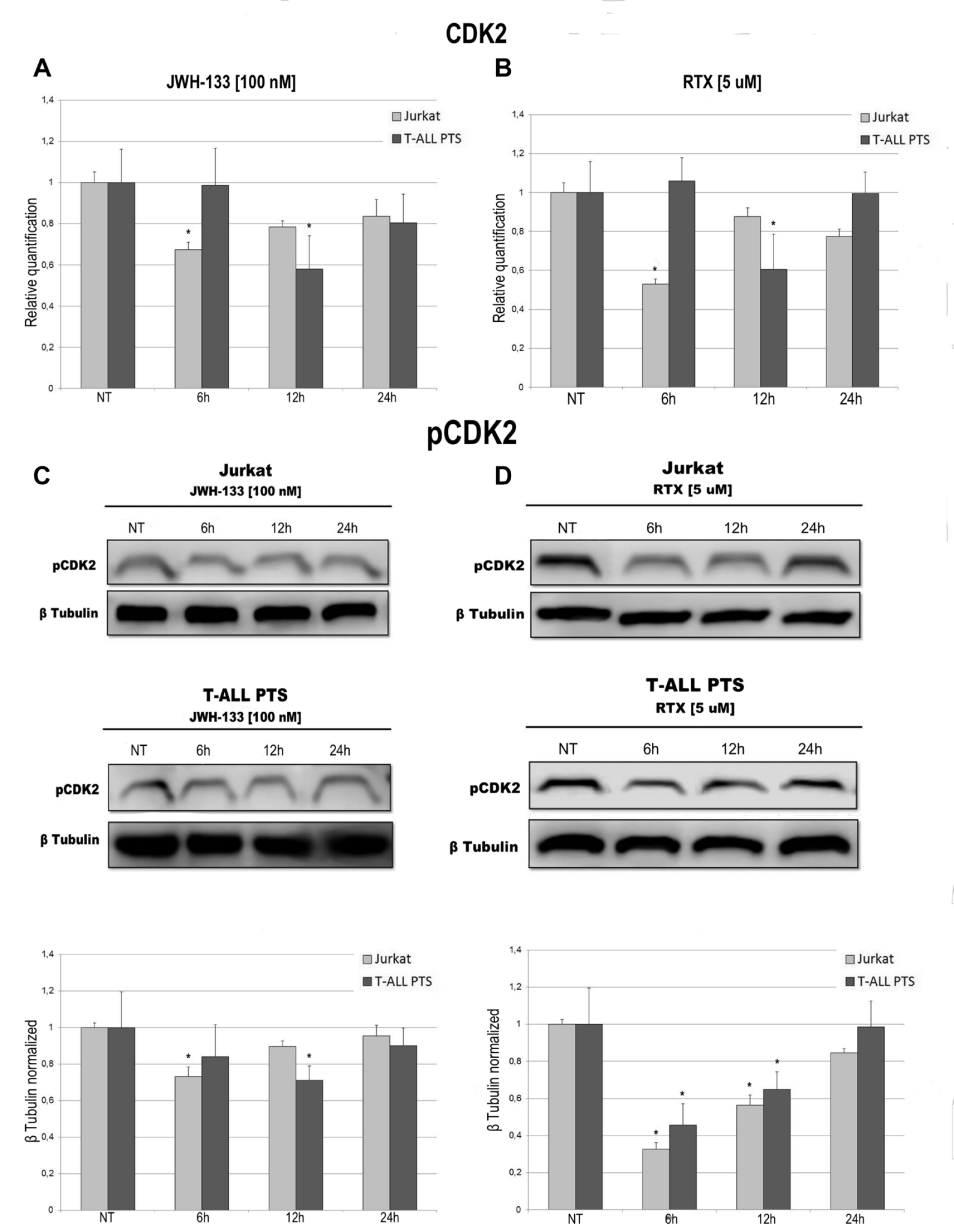
Effects of JWH-133 and RTX treatments on CDK2 mRNA expression levels and pCDK2 protein expression levels CDK2 mRNA expression levels in Jurkat cell line and in T-ALL patients’ lymphoblasts derived from 4 patients were determined by Q-PCR after JWH-133 [100 nM] (**A**) and RTX [5 uM] (**B**) treatments at 6 h, 12 h and 24 h of exposure. Results were normalized for the housekeeping gene β-actin and were showed as mean ± SD of three independent experiments for Jurkat cells (performed in technical triplicate), and as mean ± SD of four single experiments on each one of the 4 patients (performed in technical triplicate). A *t*-test has been used to evaluate statistical differences in CDK2 expression among groups. ^*^indicates *p* ≤ 0.05 compared to the untreated control (NT). pCDK2 protein expression levels in Jurkat cell line and in T-ALL patients’ lymphoblasts, were determined by Western Blot, starting from 15 μg of total lysates after JWH-133 [100 nM] (**C**) and RTX [5 uM] (**D**) treatments after 6 h, 12 h and 24 h of exposure. Images show the most representative blots. The proteins were detected using Image Studio Digit software and the intensity ratios of immunoblots compared to that of untreated control, taken as 1 (arbitrary unit), were quantified after normalizing with respective loading controls for the housekeeping protein β-tubulin. The graphs represent the relative quantification for pCDK2 expression as mean ± SD of two independent experiments for Jurkat cells and as mean ± SD of four single experiments on each one of the 4 patients. A *t*-test has been used to evaluate the statistical differences in protein expression levels. ^*^indicates *p* ≤ 0.05 compared to the untreated control (NT).

To evaluate the possible role of EC/EV drugs on cell cycle progression we performed a specific assay on the Muse Cell Analyser. Cells were incubated for 6 h, 12 h, 24 h and 48 h after exposure to JWH-133 [100 nM], RTX [5 μM] or untreated. The Muse cell Analyser automatically displayed the percentage of cells in G0/G1, S and G2/M phases of the cell cycle (Table 2). In Jurkat cells, while already at 12 h there is a marked reduction of cells in S phase, at 24 h we observe an increase of cells in G0/G1. This increase is still present when performing the analysis at 48 h, demonstrating clearly that those cells did not proceed to the next phases of the cell cycle (Table 2A) which indicates an impairment in cell cycle progression caused by EC/EV agonists. In T-ALL patients cells (Table 2B) the results show the same pattern but are not statistically relevant, probably due to the samples heterogeneity or slower cell replication time. To confirm what observed in the Cell Cycle Assay we evaluated the expression levels of CDK2 both at mRNA levels and the phosphorylated protein. JWH-133 [100 nM] and RTX [5 μM] reduced the expression of this important cycline for cell cycle progression, in a statistically significant manner, even if at different time points (6 h for Jurkat and 12 h for T-ALL patients cells) (Figure 6A, 6B). By Western Blot we analysed the expression of the active form of this protein (phosphorylated) and confirmed the result obtained on mRNA (Figure 6C, 6D).

**Table 2:** Cell Cycle progression analysis of Jurkat cell line and T-ALL patients’ lymphoblasts treated with JWH-133 and RTX

**A**			
**Jurkat**	**G0/G1(%)**	**S(%)**	**G2/M(%)**
NT	38.5 ± 3.5	28.1 ± 4.4	33.1 ± 3.8
JWH-133 [100 nM] 6 h	37.5 ± 3.2	45.3 ± 3.6^*^	16.7 ± 3.9^*^
JWH-133 [100 nM] 12 h	37.0 ± 4.4	14.8 ± 4.5^*^	48.2 ± 3.8^*^
JWH-133 [100 nM] 24 h	48.2 ± 3.1^*^	12.6 ± 2.8^*^	39.1 ± 2.7
JWH-133 [100 nM] 48 h	46.8 ± 3.3^*^	13.1 ± 2.8^*^	39.9 ± 2.4
RTX [5 μM] 6 h	42.3 ± 3.8	36.8 ± 3.4^*^	20.9 ± 3.7^*^
RTX [5 μM] 12 h	39.1 ± 4.5	14.3 ± 3.9^*^	46.6 ± 3.9^*^
RTX [5 μM] 24 h	47.4 ± 4.3^*^	12.6 ± 3.2^*^	40.0 ± 3.5
RTX [5 μM] 48 h	49.6 ± 4.6^*^	11.6 ± 3.4^*^	38.5 ± 3.6
**B**			
**T-ALL T PTS**	**G0/G1(%)**	**S(%)**	**G2/M(%)**
NT	26.64 ± 4.5	12.8 ± 4.9	60.56 ± 4.7
JHW-133 [100 nM] 6 h	23.3 ± 4.1	12.0 ± 4.6	64.7 ± 4.2
JHW-133 [100 nM] 12 h	26.1 ± 4.9	11.8 ± 5.2	62.1 ± 5.1
JHW-133 [100 nM] 24 h	26.0 ± 5.3	10.7 ± 4.8	63.3 ± 4.6
JHW-133 [100 nM] 48 h	26.9 ± 5.1	10.1 ± 4.6	63.0 ± 4.5
RTX [5 μM] 6 h	35.7 ± 4.9	11.7 ± 5.1	52.6 ± 5.2
RTX [5 μM] 12 h	27.4 ± 4.6	10.3 ± 5.6	62.3 ± 5.7
RTX [5 μM] 24 h	27.4 ± 4.8	9.3 ± 5.6	63.3 ± 5.3
RTX [5 μM] 48 h	27.1 ± 4.9	9.1 ± 5.8	63.8 ± 5.1

Jurkat cell line and T-ALL patients’ lymphoblasts were cultured and treated with JWH-133 [100 nM] and RTX [5 uM], for 6 h, 12 h , 24 h and 48 h. Cells were harvested, fixed and then incubated with Muse Cell Cycle reagent for 30 minutes and analyzed on “MUSE Cell Analyzer”. The percentage of cells accumulated at different phases of the cell cycle (G0/G1 phase, S phase and G2/M phase) are shown. The results are presented as the mean percentage ± SD of three independent experiments for Jurkat cells (A) and 4 single experiment on each one of the 4 patients (B) and were analyzed by one-way analysis of variance (ANOVA). ^*^indicates *p* ≤ 0.05 compared to the untreated control (NT).

## DISCUSSION

T-cell Acute Lymphoblastic Leukemia (T-ALL) represents about 10–15 % of pediatric ALL cases but it has a worse outcome compared to B-lineage ALL, especially when the patient presents poor response to prednisone therapy [5, 7, 8]. For this reason new therapeutic approaches are needed.

Cannabinoids have been shown to induce apoptosis in human leukemia and lymphoma cell lines via CB2, the cannabinoid receptor normally expressed in the immune system [14–16, 33, 34]. TRPV1 has been found to be functionally expressed in CD4^+^ T cells and it contributes to T cell receptor (TCR)-induced Ca^2+^ channel influx [35].

In the present study we analyzed the therapeutic potential of two EC/EV drugs, JWH-133 (a potent CB2 selective agonist) and RTX (an analog of capsaicin, a vanilloid agonist), in primary cell cultures obtained from 4 patients affected with T-ALL and in Jurkat cell line.

The treatments were carried out at different incubation times, but the most significant effects were observed after 6 hours of drug administration, especially in the Jurkat cell line and mostly after 12 hours in T-ALL patients’ primary cell cultures, since these cells duplicate much slower than Jurkat. Many signaling pathways involved in apoptosis, cell cycle progression and proliferation are usually dysregulated in cancer cells [6]. In this study, we demonstrated that stimulation of EC/EV system induces apoptosis in both cultured T-ALL patient cells and in Jurkat cells. Firstly we demonstrated an increase in apoptosis in the cells studied. Moreover considering the central role of Caspase-3 in executing apoptosis [36], and the observation that several cancer cells exhibit altered Caspases-3 expression [37, 38], we also examined the expression levels of Caspase-3 in our samples, which resulted increased after EC/EV stimulation. To better understand the biological mechanism through which these compounds could increase apoptosis we also evaluated the pro-apoptotic protein Bax and the anti-apoptotic Bcl2 demonstrating an increased ratio which indicates a tendency to induce the apoptotic cascade.

On the other hand we investigated the effect of JWH-133 and RTX on the cell survival pathways. It is well established that Mitogen-Activated Protein Kinase (MAPK) cascades, especially those involving extracellular signal-regulated kinase (Erk) 1/2 activated by MAPK/Erk kinase (MEK) 1/2 dual-specificity protein kinases, promote cancer cell survival [39, 40]. The constitutive activation of PI3K/Akt signaling pathway is a very common event in T-ALL and is critical for leukemic cell viability. PI3K/Akt pathway hyperactivation appears to result mainly from post-translational inactivation of the Phosphatase and Tensin homolog (PTEN) protein, which is the main negative regulator of PI3K/Akt pathway [39, 41–43]. The activation of PI3K/Akt is also associated with Notch1 activation. Notch 1 transcriptionally represses PTEN. A significant subset of newly diagnosed T-ALL patient samples are known to present inactivating PTEN gene mutations. These mutations may collaborate with PTEN post-translational inactivation to maximize its functional deficiency, thus contributing to increase PI3K/Akt activation and finally to leukemia resistance to chemotherapy [44–46]. In our study we demonstrated a downregulation of ERK and AKT especially at protein level in their active forms and also a decrease of Notch-1 mRNA level, together with an overexpression of PTEN. Studies revealed that down regulation of Notch-1 could induce G(0)/G(1) cell cycle arrest and apoptosis. The effects might be mediated by regulating the Cycline dependent Kinases 2 (CDK2) expression and the Akt signaling [47, 48]. For this reason we performed also a Cell Cycle Progression Assay and examined the expression levels CDK2 at mRNA and protein level.

In tumors, cell cycle dysregulation caused an abnormal cell growth. More than 90% of human cancers have been found with the alterations of cyclin-dependent kinases (CDKs) which were most related with G1 phase [49].The transition from G0/G1 to S phase is responsible for initiation and completion of DNA replication. In the present study, in Jurkat cells we observed first a reduction of cells in S phase and subsequently a significant accumulation in G0/G1-phase compared with cancerous untreated cells. To further investigate the molecular basis by which CB2 and TRPV1 agonists inhibited the G0/G1 transition in T-ALL, we also analyzed the expression of CDK2 mRNA and the active form of the protein pCDK2. This protein has been found downregulated by EC/EV ligands which confirm that RTX and JWH-133 might trigger the progression of cell cycle in T-ALL and may result in the blockage of cell division, cell death, and/or apoptosis.

Moreover, in our study we demonstrated a significant upregulation of p53 by RTX and JWH-133. P53 is a tumor suppressor that is lost or inactivated in the majority of tumors [50]. P53 protein is a tumor suppressor that serves as a genomic guardian to maintain a dynamic balance between cell growth and cell arrest in response to genomic stress [51]. Mutations and/or deletions in p53 predict a poorer outcome in T-ALL patients [52, 53]. The activation of P53 can be beneficial for cancer treatment outcome to contribute to more efficient tumor cell killing by restoring p53 function to the level of a normal tissue in those patients who do not suffer from a genetic mutation on that gene.

In conclusion we demonstrated a downregulation of genes involved in cell cycle progression and proliferation, and upregulation of genes involved in apoptosis together with cell cycle arrest in Jurkat cells, following administration of selective agonist of CB2 and TRPV1 receptors.

Taken together, these data show an important switch toward the programmed cell death in the cells studied following EC/EV stimulation. Other studies have been conducted using cannabinoids in Jurkat cell line but we used also patients’ derived lymphoblasts and in prospective of a clinical application, we chose two drugs selectively acting on TRPV1 and CB2 receptors and so probably without psychotropic effects. Further studies, combining the compounds used in this study with current conventional T-ALL therapy, and *in-vivo* studies on animal models, are required to provide a better insight on the importance of CB2 and TRPV1 in leukemia, to investigate the precise molecular mechanism of these antitumor activities and whether this co-administration could be effectively applied in clinical practice. In conclusion, our results strongly support the potential of EC/EV system as new therapeutic target in T-ALL, having demonstrated its capacity to interfere in proliferation and apoptosis of cancer cells by increasing/decreasing target genes involved in proliferation and apoptosis.

## MATERIALS AND METHODS

### Patients

Four Caucasic T-ALL patients referred to our center were included in this study. A girl of 5 years old, and three boys of 9, 10 and 12 years old at the diagnosis. These patients did not present cytogenetic alterations. A signed study subject informed consent was obtained from all subjects with the approval of Ethics Committee of the University of Campania “Luigi Vanvitelli” and in compliance with national legislation and the Code of Ethical Principles for Medical Research Involving Human Subjects of the World Medical Association (Declaration of Helsinki)

### Cell cultures

Jurkat cell line has been purchased from Sigma-Aldrich. The patient's Lymphoblasts were obtained

from peripheral blood mononuclear cells (PBMCs) isolated by centrifugation over Histopaque 1077 density gradient (Sigma). Jurkat cell lines and patient's Lymphoblasts were cultured in RPMI medium with 10% fetal bovine serum (FBS), supplemented with 100 U/ml penicillin (Gibco), 100 U/ml streptomycin (Gibco) and 2 mM L-glutamine (Euroclone). Cells were cultured at 37° C in a humidified atmosphere with 5% CO_2_.

### Drugs and treatments

Resiniferatoxin (RTX- Potent analog of capsaicin, that is an agonist at vanilloid receptors) and JWH-133 (Potent CB2 selective agonist. Approx. 200-fold selective over CB1 receptors) were purchased from Tocris Bioscience (Bristol, UK). The powder was dissolved in dimethyl sulfoxide (DMSO) at a concentration of 50mM for JWH-133. RTX was dissolved in DMSO to a stock solution of 100 mM. DMSO final concentration on cell cultures was 0.01%. Stock solutions were aliquoted and kept at −80° C for long-term storage. Jurkat and primary cell lines were treated with EC/EV compounds alone at concentrations of RTX [5 uM] and JWH-133 [100 nM]. Non-treated cultured cell lines were maintained in incubation media during the relative treatment time with and without vehicle (DMSO 0.01%).

### Annexin, count and viability

Apoptosis has been evaluated by a fluorometric assay on the Muse cell analyser machine with the “Cell dead and Annexin V Assay Kit”. Test was performed after 6 h, 12 h and 24 h of EC/EV compounds exposure. EC/EV active compounds were added alone at the following concentrations: JWH-133 [100 nM], RTX [5 uM]. The Muse™ Annexin V & Dead Cell Assay utilizes Annexin V to detect phosphatidylserine (PS) on the external membrane of apoptotic cells. A dead cell marker, is also used as an indicator of cell membrane structural integrity,7-amino-actinomycin D (7-AAD). Briefly, 100 μL of a cell suspension (1 × 10^4^ cells/mL) was mixed with100 μL of Muse™ Annexin V & Dead Cell Reagent and incubated for 20 minutes at room temperature in dark. The results, automatically displayed, were analyzed with “Muse 1.4 Analysis” software for data acquisition and analysis.

### Cell cycle assay

Cell cycle has been evaluated by a fluorometric assay on the Muse cell analyser machine with the “Cell Cycle Assay Kit”. Test was performed after 6 h, 12 h and 24 h of EC/EV compounds exposure. EC/EV active compounds were added alone at the following concentrations: JWH-133 [100 nM], RTX [5 uM]. The Muse™ Cell Cycle Assay utilizes a propidium iodide (PI)-based staining of DNA content to discriminate and measure the percentage of cells in each cell cycle phase (G0/G1, S, and G2/M). Briefly, Jurkat and Lymphoblasts cells were fixed in 70% ice-cold ethanol for at least 30 minutes, washed and incubated with Cell Cycle reagent for 2 hours at room temperature in dark. The results, automatically displayed, were analyzed with “Muse 1.4 Analysis” software for data acquisition and analysis.

### Total RNA extraction and reverse transcription quantitative polymerase chain reaction (RTqPCR)

Following treatment with JWH-133 [100 nM] and RTX [5 uM] and incubation for 6 h, 12 h and 24 h at 37° C with 5% CO_2_, OS cells were harvested. Cells without treatment served as the control group. The total RNA was extracted using Quiazol^®^ (Quiagen) following the manufacturer's instructions. EasyScript™ cDNA Synthesis Kit (abm) was used to synthesize from approximately 1000 ng mRNA, the first strand cDNA. The transcript levels of AKT serine/threonine kinase (AKT), mitogen-activated protein kinase 1 (MAPK1/ERK), phosphatase and tensin homolog (PTEN), Notch-1, cyclin dependent kinase 2 (CDK2), tumor protein p53 (p53) and Caspase-3 (CASP3) were detected by RT-qPCR using a CFX96 Real-Time PCR system (Bio-Rad) using I-Taq Universal SYBR^®^ Green Master Mix (Bio-Rad). The cycling conditions were 10 min at 95° C (initial denaturation) followed by 40 cycles of 15 sec at 94° C (denaturation) and 1 min at 68° C (annealing/extension/data collection). The β-Actin gene served as the reference gene for the normalization of the real-time PCR products. The PCR primers used to detect each gene were designed using Primer 3 program and synthesized by Sigma Aldrich (CB2_F 5′-AAGGCTGTCTTCCTGCTGAA-3′, CB2_R 5′- CACAGAGGCTGTGAAGGTCA-3, TRPV1_F 5′-CTG CAGAAGAGCAAGAAGCA-3′, TRPV1_R 5′-ATGGC TTTCAGCAGACAGGT-3′, AKT_F 5′-GCTCACCCAG TGACAACTCA-3′, AKT_R 5′- CCCAGCAGCTTCAG GTACTC-3′, ERK_F 5′- GTGACCTCAAGCCTTCCA AC-3′, ERK_R 5′- TTCTGGAGCCCTGTACCAAC-3′, PTEN_F 5′-TCCACAAACAGAACAAGATGCT-3′, PTEN_R 5′-GGTTTCCTCTGGTCCTGGTA −3′, Notch1_F 5′-TCCTTCTACTGCGAGTGTCC–3′, Notch1_R 5′- TCG TTACAGGGGTTGCTGAT −3′, CDK2_F 5′- TCACTGGC ATTCCTCTTCCC −3′, CDK2_R 5′- ACCCGATGAGAA TGGCAGAA −3′, p53_F 5′- CCTCACCATCATCACA CTGG −3′, p53_R 5′- TTGCGGAGATTCTCTTCCTC −3′, Caspase3_F 5′-TTGTGGAATTGATGCGTGAT-3′, Caspase 3_R 5′-TGGCTCAGAAGCACACAAAC-3′, β-Actin_F 5′-GCGAGAAGATGACCCAGATC-3′, β-Actin_R 5′-GGA TAGCACAGCCTGGATAG-3′). The linearity and efficiency of the assays were tested over dilutions of input cDNA spanning five orders of magnitude. Assays were performed in technical triplicate, for three times on Jurkat cells (displayed as mean ± SD) and one time for each one of the 4 patients (displayed as mean ± SD). The dissociation curve analysis of amplification products was performed at the end of each PCR reaction to confirm the specificity of the amplification. The 2^-ΔΔCt^ method was used to analyze the data and obtain the relative gene expression levels compared to the controls.

### Western blotting

Proteins were extracted from treated and non-treated primary T-Cell ALL patient's and Jurkat cell line using RIPA Lysis Buffer (Millipore) and following the manufacturer's instructions. pAKT, pERK Bcl-2, Bax, Pro-Caspase 3, and pCDK2 proteins were characterized in total lysates from cell cultures by Western blotting. Membranes were incubated overnight at 4° C with rabbit polyclonal anti pAKT antibody (1:200 dilution; Santa Cruz), mouse polyclonal anti pERK (1:200 dilution; Santa Cruz), mouse polyclonal anti Bcl-2 (1:500 dilution; Santa Cruz), rabbit polyclonal anti Bax antibody (1:1000 dilution; Cell Signaling), mouse polyclonal anti-Pro-Caspase 3 antibody (1:1000 dilution; Abcam), rabbit polyclonal anti pCDK2 (1:1200 dilution; Abcam). For determination of Bax and Bcl-2 ratio, antibodies against both proteins were used subsequently on the same membrane. Reactive bands were detected by chemiluminescence (Immobilon western Millipore) on a C-DiGit^®^ Blot Scanner (LI-COR Biosciences). A mouse polyclonal anti β-Tubulin antibody (1:1000 dilution; Sigma) was used to check for comparable protein loading and as a housekeeping protein. We performed two experiments on Jurkat cells (displayed as mean ± SD) and single experiments on each one of the 4 T-ALL patients’ samples (displayed as mean ± SD). Images were captured, stored, and analyzed using “Image studio Digits ver. 5.0” software.

### Statistical analysis

Results are expressed as means ± S.D. The experiments were run in technical triplicate for Real Time PCR. When performing Western Blotting, two times for Jurkat cells and displayed as mean ± SD, single experiments on each one of the 4 T-ALL patient's and displayed as mean of all of them ± SD. Statistical analyses on molecular and biochemical data were performed using Student's *t* test to evaluate differences between quantitative variables. Statistical analyses on apoptosis and cell cycle data were performed using one-way analysis of variance, ANOVA (StatGraphics Centurion XV.II Software. Adalta, Arezzo, Italy; Statpoint Technologies Inc., VA). A p value less than 0.05 was considered statistically significant.

## SUPPLEMENTARY MATERIALS FIGURES AND TABLES


