# Supplementary material for: Effects of CB2 and TRPV1 receptors’ stimulation in pediatric acute T-lymphoblastic leukemia
Source: Oncotarget. 2018 Apr 20;9(30):21244–58. doi: 10.18632/oncotarget.25052 (PMC5940388; doi:10.18632/oncotarget.25052)
Supplement: Supplementary file 1 [file oncotarget-09-21244-s001.pdf]

Effects of CB2 and TRPV1 receptors' stimulation in Pediatric Acute T-lymphoblastic Leukemia

SUPPLEMENTARY MATERIALS

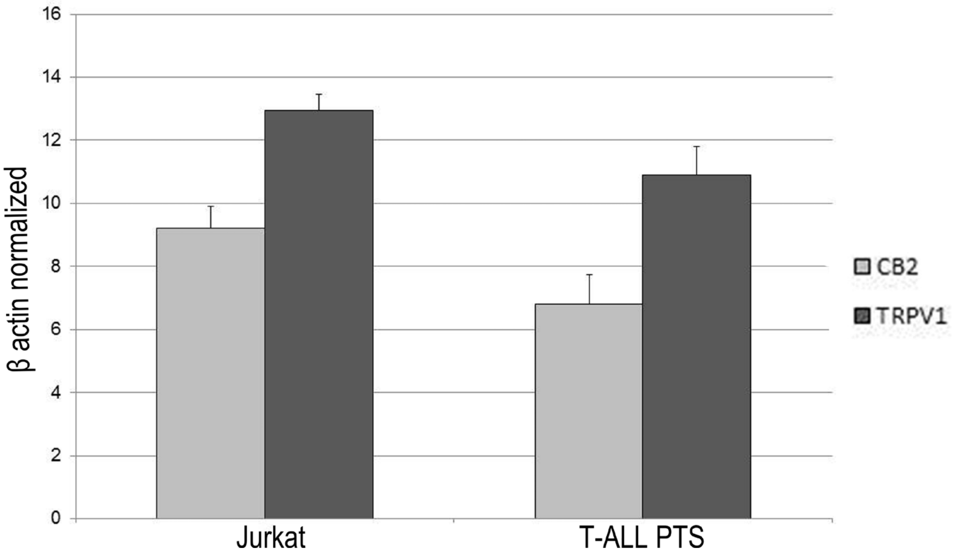

Supplementary Figure 1: EC/EV receptors' expression in Jurkat cell line and in T-ALL patients' lymphoblasts. Jurkat cell line and T-ALL patients' lymphoblasts, obtained from 4 different patients, express the Cannabinoid receptor type 2 (CB2) and the Transient Receptor Potential Vanilloid type-1 (TRPV1). Data have been revealed by Q-PCR, starting from 1000 ng of total mRNA used in a Reverse Transcriptase reaction. Ct values were normalized for the housekeeping gene β-actin and were showed as mean ± SD of three independent experiments for Jurkat cells and as mean ± SD of one experiment, performed in triplicate, for each one of the 4 different patients.

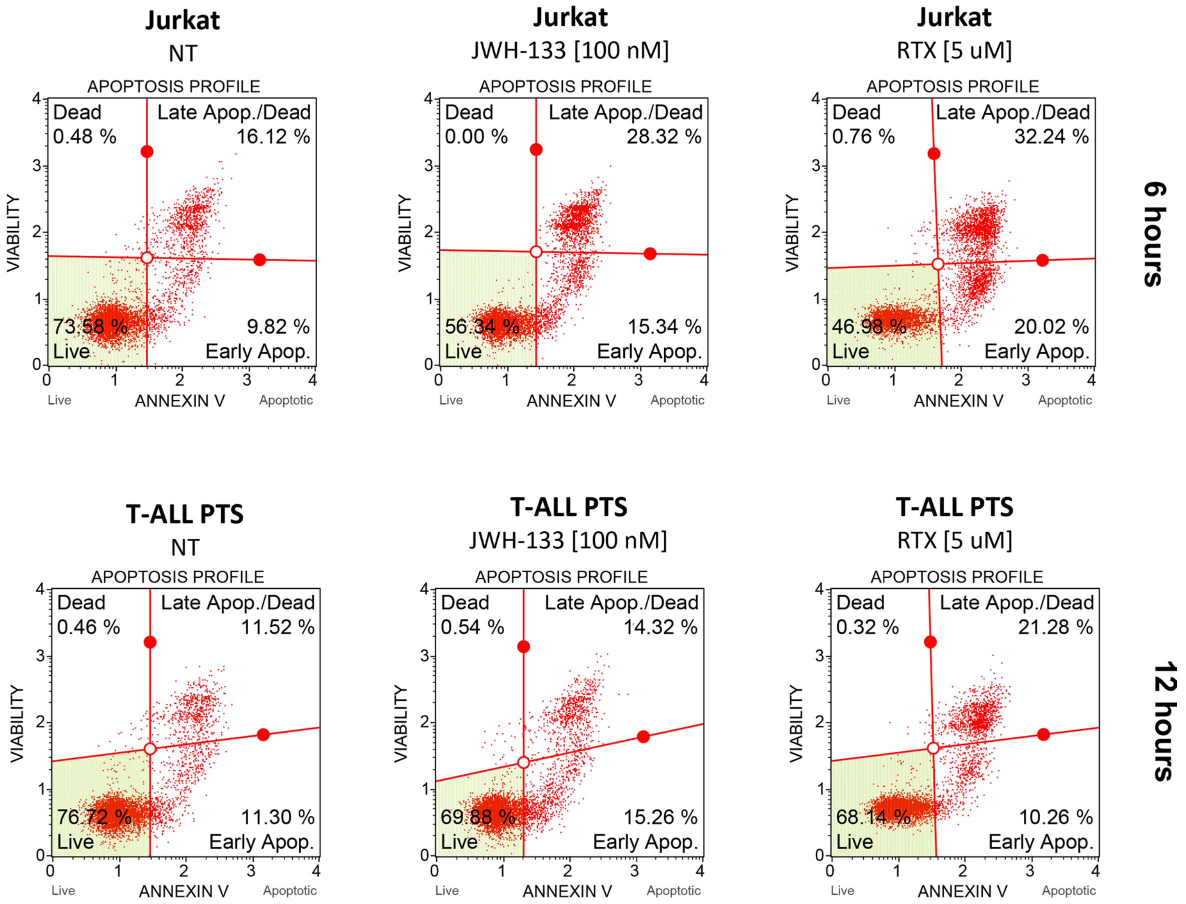

Supplementary Figure 2: Representative selection of Apoptosis Assay's Dot Plots of Jurkat cells and T-ALL Patients' lymphoblasts after JWH-133 and RTX treatments. The panels show more representative cytofluorimetric scatter plots of Apoptosis Assay performed on Jurkat cell line and T-ALL patients' lymphoblasts. Apoptosis Assay was performed using "Cell dead and Annexin V Assay Kit" (Millipore) on a MUSE Cell Analyzer, following the manufacturer's protocol. The results were analyzed with "Muse 1.4 Analysis" software.
